# Scattering of Metal Colloids by a Circular Post under Electric Fields

**DOI:** 10.3390/mi14010023

**Published:** 2022-12-22

**Authors:** José Eladio Flores-Mena, Pablo García-Sánchez, Antonio Ramos

**Affiliations:** 1 Facultad de Ciencias de la Electrónica, Benemérita Universidad Autónoma de Puebla, Av. San Claudio y 18 Sur, San Manuel, CU. FCE2, Puebla 72570, Mexico; 2 Departamento Electrónica y Electromagnetismo, Facultad de Física, Universidad de Sevilla, Avda. Reina Mercedes s/n, 41012 Sevilla, Spain

**Keywords:** AC electrokinetics, microfluidics, induced-charge electroosmosis, dielectrophoresis

## Abstract

We consider the scattering of metal colloids in aqueous solutions by an insulating circular post under the action of an AC electric field. We analyze the effects on the particle of several forces of electrical origin: the repulsion between the induced dipole of the particle and its image dipole in the post, the hydrodynamic interaction with the post due to the induced-charge electroosmotic (ICEO) flow around the particle, and the dielectrophoresis arising from the distortion of the applied electric field around the post. The relative influence of these forces is discussed as a function of frequency of the AC field, particle size and distance to the post. We perform numerical simulations of the scattering of the metal colloid by the insulating circular post flowing in a microchannel and subjected to alternating current electric fields. Our simulation results show that the maximum particle deviation is found for an applied electric field parallel to the flow direction. The deviation is also greater at low electric field frequencies, corresponding to the regime in which the ICEO’s interaction with the post is predominant over other mechanisms.

## 1. Introduction

Electric fields are commonly used in microfluidic devices to manipulate particles with the objective of segregating them [1]. Particle separation is often employed in the analysis of biological samples containing, for example, cells, vesicles, and macromolecules. In particular, deterministic lateral displacement (DLD) devices with electric fields have been studied for particle separation [2,3]. In these devices, the characteristics of the particle’s interaction with an insulating post is central for the efficiency of the separation mechanism. For example, Ho et al. have recently demonstrated the segregation of micro- and nanoparticles based on their surface charge using DLD devices [4]. Additionally, Calero et al. have shown that the combination of AC and DC electric fields can give rise to the fractionation of submicrometer particles within arrays of 20 micron diameter posts [5].

In this work, we study the scattering of colloidal metal particles by a cylindrical insulating post. The particles are suspended in an electrolyte that flows in a microchannel with a prescribed velocity and encounter a post of circular shape. The post distorts the flow streamlines, which recover their original shape past the post. In the absence of an electric field, colloidal particles follow the streamlines, and, therefore, their motion remains unaltered after the post is left behind. Notice that this is the case for particle trajectories not very close to the post, while for trajectories close to the post, there are non-hydrodynamic, short-range interactions that lead to some deviations of the order of the particle diameter [6]. These hard-wall interactions constitute the physical mechanism for particle deviation of the original DLD devices. During the 1990s, experimental and theoretical works on the scattering of a colloidal particle by another particle were performed as a way of studying contact forces [7,8,9]. If an electric field is applied, the distortion of the electric field lines caused by the cylinder gives rise to a non-homogeneous field that leads to dielectrophoretic (DEP) forces on the particles, i.e., a net electrical force on the particle-induced dipole. The application of DEP within arrays of posts for particle concentration [10,11,12] and particle filtration [13,14] has been extensively demonstrated in microfluidics experiments. It has recently been shown how to fabricate and use arrays of metal posts for the DEP deviation of microparticles within microchannels [15]. Besides the DEP force, the particle also undergoes an electrical wall repulsion arising from the image dipole. Furthermore, AC electric fields induce quadrupolar electroosmotic flows around metal colloids that can give rise to particle-wall repulsion, as observed in electrophoresis experiments for nonmetal colloids [16] and particle-particle interactions [17,18]. All these electrical interactions are able to modify particle trajectories from the original streamlines. We use numerical simulations to study the interplay of these phenomena on the scattering due to the post. The main novelty in these simulations is the interaction with the post due to the quadrupolar electroosmotic flows induced on the particles. For example, we demonstrate that the repulsion due to these flows is stronger than the particle repulsion with its image dipole. The latter interaction decays with distance as $r^{-4}$, while the repulsion due to the quadrupolar electrosmostic flows does so as $r^{-2}$. In addition to its fundamental interest, these results will find applications in the design of microfluidic devices that combine obstacles and/or constrictions with electric fields for particle separation and fractionation [10,19].

The paper is organized as follows. First, we describe the different interactions that we consider between a colloidal metal particle and an insulating post. The relative influence of these forces is discussed as a function of frequency of the AC field, particle size and distance to the post. Later, we present the numerical results obtained with the software Comsol Multiphysics for the particle trajectories for different parameters and draw some conclusions about the most interesting set of parameters.

## 2. Analysis of the Physical Problem

**Figure 1:**
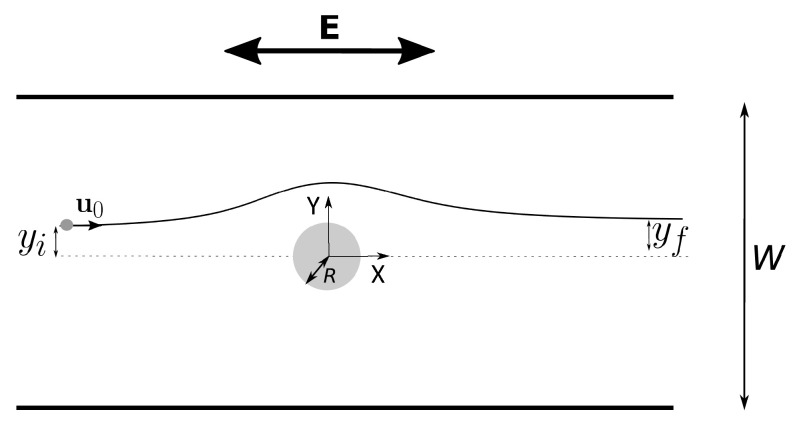
Schematics of the problem. Particles enter the channel from the left side driven by the fluid flow with velocity $u_{0}$. There is an ac electric field applied in the system. The particle trajectories are affected by the presence of an insulating post. The particle-post interaction arises from different mechanisms of electrical origin.

Let us consider a small metal particle with radius *a* in a microchannel being dragged by the flow of an electrolyte with velocity $v_{0}$. The particle approaches a cylindrical post of radius *R* made of an insulating material (see Figure 1). For simplicity, we will assume that the cylinder is infinitely long. The application of an electric field within the liquid, E, gives rise to an interaction of the particle with the post, which results in a deviation of the former with respect to its original trajectory. We place the cylinder in the middle of a domain where we impose periodic boundary conditions on the upper and lower planes. Therefore, we consider that the particle approaches one of the cylinders of an infinite row perpendicular to the flow direction. The particle-post interaction arises from the combination of the following mechanisms: particle repulsion with its image dipole, ICEO repulsion from insulating walls, and dielectrophoresis and dipolophoresis.

### 2.1. Particle Repulsion with Its Image Dipole

The electric field induces an electric dipole moment on the particle, which, in turn, is repelled from the post. This repulsion can be described as the interaction of the electric dipole with its image dipole due to the proximity of an insulating wall. Let us consider that the particle is in front of a plane wall placed at z=0 and that there is an AC field with angular frequency ω at the particle position, such as E(t)=Re[(Exx^+Ezz^)exp(iωt)]. In this situation, the particle acquires an induced electric dipole p=Re[4πεa3α(Exx^+Ezz^)exp(iωt)], where ε is the electrical permittivity of the electrolyte, and α is the complex non-dimensional polarizability of the particle (also known as the Clausius–Mossoti factor [20]). Thus, the time-averaged repulsion force of the particle dipole at z=h with the wall at z=0 is given by:

(1) $$F_{p\text{-}p}=\frac{3\pi \varepsilon a^{6}\alpha \alpha^{*}\left(2E_{z}^{2}+E_{x}^{2}\right)}{8h^{4}}\hat{z}$$

where ε is the medium permittivity, and ${}^{*}$ indicates the complex conjugate.

The polarizability of a metal sphere in an electrolyte is determined by the charging of the electrical double layer (EDL) induced at the particle-electrolyte interface and can be written as [21]:

(2) $$\alpha =\frac{i(\omega /\omega_{\mathrm{DL}})-1}{i(\omega /\omega_{\mathrm{DL}})+2}$$

where $\omega_{\mathrm{DL}}$ is the reciprocal of the RC charging-time of the sphere EDL ($\omega_{\mathrm{DL}}=\sigma /aC_{\mathrm{DL}}$, with σ being the liquid conductivity and $C_{\mathrm{DL}}$ being the specific surface capacitance of the EDL). Since the particle is immersed in a liquid with viscosity η and inertia is negligible for micron-size particles, this force is balanced by the Stokes drag on a sphere, i.e., $F_{\mathrm{drag}}=6\pi \eta au$, with *u* being the particle velocity. Thus, the particle repels from the wall with a velocity given by:

(3) $$u_{p\text{-}p}=\frac{\varepsilon a^{5}\left(2E_{z}^{2}+E_{x}^{2}\right)}{16\eta h^{4}}\left(\frac{1+{\left(\omega /\omega_{\mathrm{DL}}\right)}^{2}}{4+{\left(\omega /\omega_{\mathrm{DL}}\right)}^{2}}\right)\hat{z}$$

Although the expression above is found for a flat wall, numerical calculations in Appendix A show that it is a good approximation for the repulsion from a circular cylinder. Thus, for simplicity, in our simulations, we will use the following expression in polar coordinates for the velocity of a particle at a position $r=\rho \hat{\rho }$ with ρ>R in a reference frame centered at the cylinder axis:

(4) $$u_{p\text{-}p}=\frac{\varepsilon a^{5}\left(2E_{\rho }^{2}+E_{\varphi }^{2}\right)}{16\eta {\left(\rho -R\right)}^{4}}\left(\frac{1+{\left(\omega /\omega_{\mathrm{DL}}\right)}^{2}}{4+{\left(\omega /\omega_{\mathrm{DL}}\right)}^{2}}\right)\hat{\rho }$$

where $E_{\rho }$ and $E_{\varphi }$ are, respectively, the radial and angular components of the electric field in polar coordinates.

### 2.2. ICEO Repulsion from Insulating Walls

Another effect of the electric fields on metal particles dispersed in electrolytes is the generation of electroosmotic flows on their surface [22]. This phenomenon is commonly known as induced-charge electroosmosis (ICEO, [23]) and it is caused by the action of the electric field on the charges of the EDL that the field induces on the metal-electrolyte interface. For a spherical metal particle, this electroosmotic velocity gives rise to an axysimmetric quadrupolar flow pattern, with the axis defined by the electric field direction and with a velocity field in spherical coordinates given by [24]:

(5) $$v=U\left(\frac{(1-{\left(r/a\right)}^{2})\left(1+3\cos2\theta \right)}{2{\left(r/a\right)}^{4}}\hat{r}+\frac{\sin2\theta }{{\left(r/a\right)}^{4}}\hat{\theta }\right),$$

where *U* is the maximum slip velocity on the sphere and is given by [22]:

(6) $$U=\Lambda \frac{\left(9\varepsilon E^{2}a/4\eta \right)}{4+{\left(\omega /\omega_{\mathrm{DL}}\right)}^{2}}.$$

Here, *E* is the amplitude of the applied electric field ($E^{2}=E_{x}^{2}+E_{z}^{2}$), and Λ is a parameter that accounts for the deviation from the ideal EDL. Λ=1 in the ideal case, where all the EDL voltage contributes to the slip velocity. However, experimental observations show that Λ<1 [21]. ICEO flows around metal particles give rise to hydrodynamic interactions between them, as theoretically studied in refs. [25,26,27] and observed in experiments [17,28].

If the metal sphere is close to an insulating wall, the ICEO flow pattern gives rise to a wall-particle interaction (see, for instance, [16]). As shown by Yariv [29], a small sphere at a distance *h* over a flat wall (h≫a) moves away from it with a velocity given by $u_{\mathrm{ICEO}}=3Ua^{2}/8h^{2}$.

We will consider a more general case for the interaction with a flat wall in which the particles are small but the direction of the electric field is arbitrary. For r≫a, the velocity field (5) reduces to a stresslet with velocity

(7) $$v=\frac{Ua^{2}}{r^{2}}\left(1-3{\left(\hat{p}\cdot \hat{r}\right)}^{2}\right)\hat{r},$$

where $\hat{p}$ is a unit vector in the direction of the induced dipole (or the field at the particle position). Smart and Leighton Jr. [30] provided the drift velocity of a stresslet due to a nonslip flat wall. They used the image system of the Stokeslet given by Blake [31] to obtain the image system of the stresslet. The velocity of a particle with a stresslet given by Equation (7) at a distance *h* from a flat wall at z=0 is given by:

(8) $$u_{\mathrm{ICEO}}=\frac{3Ua^{2}}{8E^{2}h^{2}}\left[\left(E_{x}^{2}-2E_{z}^{2}\right)\hat{z}-2E_{x}E_{z}\hat{x}\right]$$

Note that the expression above can result in either attraction to or repulsion from the wall depending on the direction of the electric field, and that there is also a velocity component tangential to the wall. However, close to an insulating wall, the electric field components, tangential ($E_{x}$) and normal ($E_{z}$), satisfy $E_{z}\ll E_{x}$, and the interaction will mostly be repulsive.

Although Equation (8) is found for a flat wall, numerical calculations in Appendix B show that it is a good approximation for the particle’s interaction with a circular cylinder. Thus, we will use the following expression in the simulations of the particle trajectories in cylindrical coordinates centered at the cylinder axis:

(9) $$u_{\mathrm{ICEO}}=\frac{3Ua^{2}}{8E^{2}{\left(\rho -R\right)}^{2}}\left[\left(E_{\varphi }^{2}-2E_{\rho }^{2}\right)\hat{\rho }-2E_{\varphi }E_{\rho }\hat{\varphi }\right]$$

### 2.3. Dielectrophoresis and Dipolophoresis

The homogeneous applied electric field in the channel becomes distorted by the presence of the cylinder. Thus, the induced dipole p on the particle is subjected to a non-homogeneous electric field, and, consequently, an electrical force acts on the particle as given by $F_{\mathrm{DEP}}=p\cdot \nabla E$. The motion arising from this force is known as dielectrophoresis (DEP) [20], and its time-averaged value for an AC field can be calculated as:

(10) $$\langle F_{\mathrm{DEP}}\rangle =\pi \varepsilon a^{3}Re\left[\alpha \right]\nabla {\left|E\right|}^{2}$$

where the nondimensional polarizability α of a metal sphere immmersed in an electrolyte is given by (2), as mentioned above. The velocity induced on the sphere by this mechanism is

(11) $$u_{\mathrm{DEP}}=\frac{\varepsilon a^{2}}{6\eta }Re\left[\alpha \right]\nabla {\left|E\right|}^{2}$$

Another effect of a non-homogeneous electric field on a metal sphere is that the ICEO flows induced around the particle are not symmetrical and lead to particle motion. The combination of this mechanism and DEP is known as dipolophoresis [32,33,34]. For low frequencies of the electric field, the theoretical models show that the contributions of the ICEO and DEP to dipolophoresis are opposite and of the same magnitude, i.e., the dipolophoresis-induced particle velocity would be zero [32,35]. However, experiments with metal spheres show that the ICEO mechanism is usually negligibly small [21] (this fact can be accounted for by using a factor Λ≪1). In this way, at low frequencies ($\omega \ll \omega_{\mathrm{DL}}$), the induced velocity becomes $u_{\mathrm{DIP}}=u_{\mathrm{DEP}}\left(1-\Lambda \right)$, where the subscript DIP stands for dipolophoresis.

### 2.4. Particle Trajectory and Comparisons between Mechanisms

Taking into account the previous interactions of the particle with the post, the particle trajectory is obtained by integrating the velocity given by

(12) $$u=u_{p-p}+u_{\mathrm{ICEO}}+u_{\mathrm{DIP}}+v$$

where v is the fluid velocity. This expression is valid since inertial effects can be neglected for small particles. Additionally, we have simplified the purely hydrodynamic interaction of the particles with the post, which depends on the structure of the flow field around them. This is a key problem by itself without including the complications of the electric field, and it should be carried out in a 3D domain to accurately describe the interaction of the spherical particle with a cylinder. For example, that kind of calculation could be useful in predicting the critical diameter for particle deviation in DLD (deterministic lateral displacement) devices [36,37]. An accurate description of particle trajectories very close to the cylinder could be carried out by using the moving mesh (ALE) tool in Comsol. However, since our goal is to analyze the relative influence of the interactions arising from the application of an electric field, we have simplified the hydrodynamic interaction.

A dimensionless form of the particle velocity can be derived by using the cylinder radius *R*, a typical fluid flow velocity $v_{0}$, and a typical electric field magnitude $E_{0}$ as references. The particle velocity in cylindrical coordinates is then given by

(13) $$\begin{array}{c} \tilde{u}=\frac{N\beta^{5}{\left|\alpha \right|}^{2}}{16{\left(\tilde{\rho }-1\right)}^{4}}\left(2{\tilde{E}}_{\rho }^{2}+{\tilde{E}}_{\varphi }^{2}\right)\hat{\rho }+\frac{27N\beta^{3}\Lambda }{128{\left(\tilde{\rho }-1\right)}^{2}}\left[\left({\tilde{E}}_{\varphi }^{2}-2{\tilde{E}}_{\rho }^{2}\right)\hat{\rho }-2{\tilde{E}}_{\varphi }{\tilde{E}}_{\rho }\hat{\varphi }\right]\\ +\left(1-\Lambda \right)\frac{N\beta^{2}Re\left[\alpha \right]}{6}\tilde{\nabla }{\left|\tilde{E}\right|}^{2}+\tilde{v} \end{array}$$

where $N=\varepsilon RE_{0}^{2}/\eta v_{0}$ and β=a/R.

In our case of an insulating pillar in a microchannel and subjected to an electric field, we can approximate the electric potential for that around a single pillar in an homogeneous field. The electric potential phasor is then given by ${\tilde{\phi }}_{0}=-\left(\tilde{\rho }+1/\tilde{\rho }\right)\cos\varphi$. Thus, we can evaluate the terms in (13) and compare them. For low frequencies $\left(\omega \leq \omega_{\mathrm{DL}}\right)$, the ratio of maximum DEP velocity to the ICEO velocity is $u_{\mathrm{DEP}}/u_{\mathrm{ICEO}}=\left(128/81\right){\left(\tilde{\rho }-1\right)}^{2}/\beta$. The DEP force is negligibly small for small particles (β≪1, or a≪R) near the post ($\tilde{\rho }-1∼\beta$, or h∼a), where the repulsion with the wall is stronger. However, for $\tilde{\rho }-1∼1$ (or h∼R), DEP forces dominate.

ICEO flows vanish at high frequencies, and close to the post, the electrical interaction with the image dipole becomes relatively more important. For $\omega >\omega_{\mathrm{DL}}$ and using (13), $u_{\mathrm{DEP}}/u_{p\text{-}p}=\left(32/3\right){\left(\tilde{\rho }-1\right)}^{4}/\beta^{3}$, and we can neglect the DEP mechanism for small particles very close to the post. However, as in the case of low frequencies, DEP is the dominant force away from the wall.

Note that in this study, we do not consider short-range forces as in the DLVO theory [38]. These forces play a role when colloids are very close to each other or to a wall. As mentioned above, several works in the decade of the 1990s performed scattering experiments and simulations between colloidal particles as a way of testing surface attractive forces [7,8,9]. The effects of these forces are beyond the scope of the present work.

## 3. Numerical Simulations of the Trajectories

Numerical simulations were performed using the finite element method (COMSOL Multiphysics). We used a 2D domain consisting of a rectangle with a circle at its center representing the cylinder cross-section. To calculate the electric field, Laplace’s equation for the electric potential (ϕ) was solved:

(14) $$\nabla^{2}\phi =0$$

The solution to Equation (14) was found in two cases:

(A) *Electric field parallel to the fluid flow*. We imposed boundary conditions of zero normal current density (∂ϕ/∂n=0) at the cylinder surface and at upper and lower planes (see the geometry in Figure 1). Dirichlet boundary conditions were applied at the entrance and exit so that the applied electric field was equal to $E_{0}$.
(B) *Electric field perpendicular to the fluid flow*. We imposed boundary conditions of zero normal current density (∂ϕ/∂n=0) at the cylinder surface and at the entrance and exit. Dirichlet boundary conditions were applied at upper and lower planes.

The velocity field (v) was obtained by solving the equations for Stokes flow

(15) $$\eta \nabla^{2}v=\nabla p\;\nabla \cdot v=0$$

where *p* corresponds to the pressure field in the liquid. The boundary conditions are: zero velocity on the cylinder, symmetry conditions for upper and lower planes in Figure 1, and $v=v_{0}\hat{x}$ far from the cylinder at the entrance and the exit. We have assumed that the insulating cylinder is uncharged and, therefore, no electroosmosis or concentration polarization electroosmosis is considered [39].

Particle trajectories are obtained by the integration of Equation (13) and assuming that the initial positions of the particles are at the entrance of the domain (left side in Figure 1).

### 3.1. Electric Field Parallel to the Fluid Flow

First, we analyze the case when the electric field is applied along the direction of the flow.

**Figure 2:**
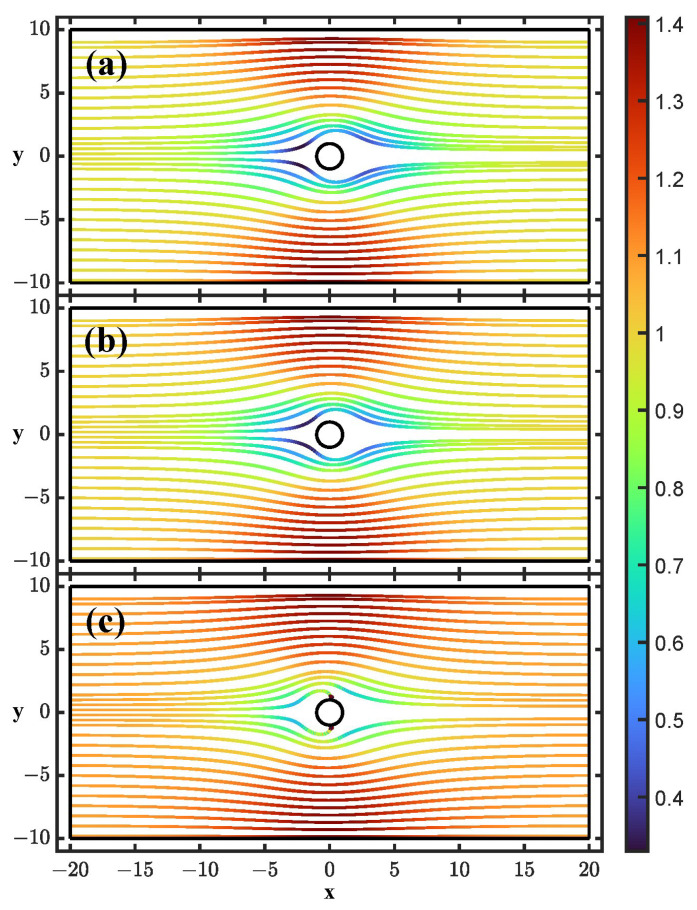
Particle trajectories for an applied electric field parallel to flow direction: (**a**) Low frequency and Λ=1. (**b**) Low frequency and Λ=0.5. (**c**) High frequency. The colors represent the magnitude of the particle velocity in units of $v_{0}$.

Figure 2 shows particle trajectories at three different conditions for N=50,β=0.2. This set of nondimensional parameters is obtained, for example, with the following values: radius of the post *R* = 10 μm, flow velocity $v_{0}$ = 100 μm/s, and electric field amplitude $E_{0}=2.67\times 10^{4}$ V/m for water at room temperature. Electric fields of this order or greater were employed in reference [40]. Figure 2 shows the particle trajectories for three different cases: (a) low frequency of the applied field and Λ=1, (b) low frequency of the applied field and Λ=0.5, and (c) high frequency. For clarification, by low and high frequency, we mean, respectively, that the frequency of the applied voltage is either much lower or much higher than $\omega_{\mathrm{DL}}$, the reciprocal of the RC time for the charging of the sphere EDL. For a 2 micron particle in a 1.5 mS/m KCl electrolyte (100 mM), we estimate $\omega_{\mathrm{DL}}/2\pi =$5 kHz.

For case (a), the dipolophoresis is zero for spherical particles, i.e., for an ideal EDL (Λ=1), the motions induced on the particle by DEP and ICEP cancel out. Therefore, particle trajectories are only affected by the reflected ICEO flow from the post and the image dipole interaction. Repulsion by the ICEO is stronger, and the dipole repulsion is only noticeable for particle trajectories that pass very close to the cylinder. It turns out that, for all cases that we studied in this work, the dipole repulsion has a negligible effect on the particles’ trajectories.

For the case (b) of low frequency and not ideal EDL with Λ=0.5, negative dielectrophoresis affects the trajectories, and the scattering from the post is a combination of ICEO repulsion and negative DEP.

It is possible to extract a function that relates the initial vertical position $y_{i}$, where the particle enters the channel, and the final exiting position $y_{f}$, where the particle leaves the channel. Cases (a) and (b) show $y_{f}>y_{i}$, that is, the vertical position at the exit is greater than the vertical position at the entrance.

Finally, for the high-frequency case shown in (c), the ICEO flow becomes negligibly small, and the particle is subjected to positive DEP since, at those frequencies, the EDL has negligible charge and the particle behaves as a conductor in a dielectric medium. As can be seen, some trajectories end on the cylinder, meaning that those particles can be trapped on the cylinder surface. This trap is where the electric field amplitude is the greatest. The particles that are not trapped show $y_{f}<y_{i}$, that is, the vertical position at the exit is smaller than the vertical position at the entrance.

### 3.2. Electric Field Perpendicular to the Fluid Flow

Now, we analyze the case when the applied electric field is perpendicular to the flow direction.

**Figure 3:**
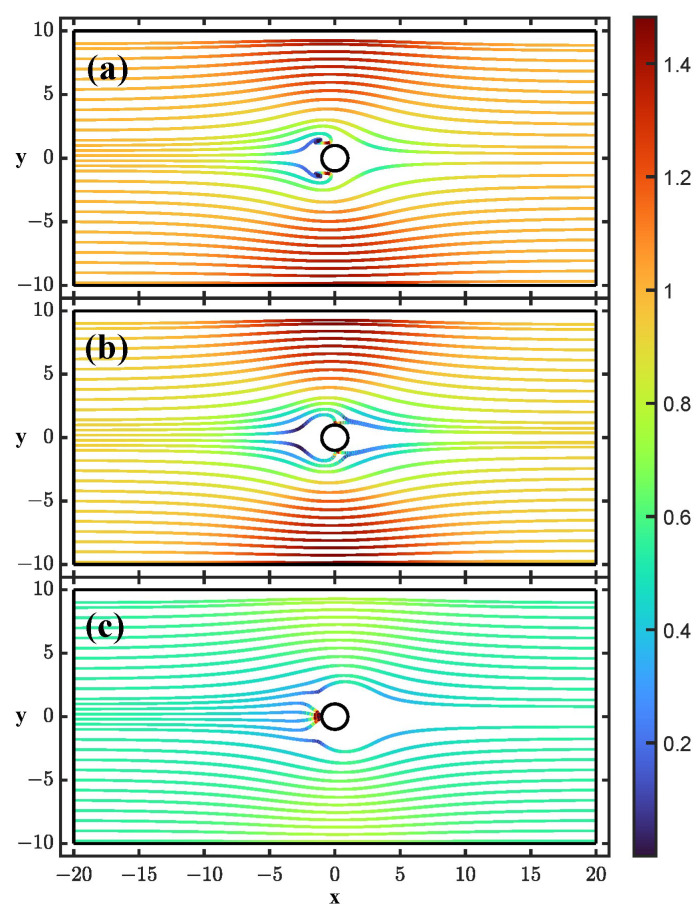
Particle trajectories for an applied electric field perpendicular to flow direction: (**a**) low frequency and Λ=1; (**b**) low frequency and Λ=0.5; (**c**) high frequency. The colors represent the magnitude of the particle velocity in units of $v_{0}$.

Figure 3 shows particle trajectories for N=50,β=0.2 at three different cases: (a) low frequency and Λ=1, (b) low frequency and Λ=0.5, and (c) high frequency.

**Figure 4:**
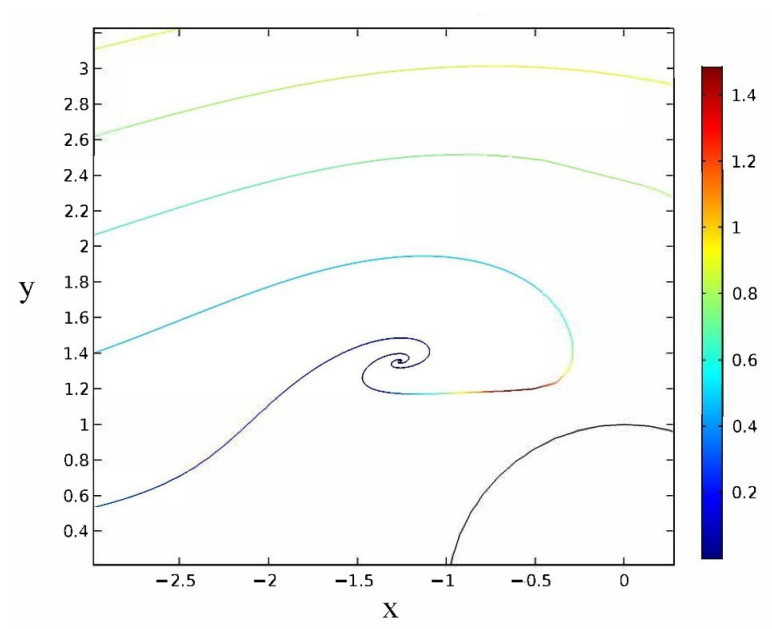
Zoom of particle trajectories for an applied electric field perpendicular to flow direction in the case of low frequency and Λ=1. The colors represent the magnitude of the particle velocity in units of $v_{0}$.

For case (a), the main effect is the ICEO interaction. Interestingly, there are trajectories where the flow velocity is balanced by the ICEO repulsion so that the particle reaches an equilibrium position on the left of the cylinder. A zoom of these trajectories is shown in Figure 4. Those trajectories that reach the exit present $y_{f}<y_{i}$, that is, the vertical positions at the exit are smaller than the vertical positions at the entrance. In this case, particles are attracted to the post because there is an important component of the electric field $E_{\rho }$, which makes the trajectories bend towards the center of the channel.

For case (b) of low frequencies and Λ=0.5, the results are similar to the previous case, but without equilibrium points. DEP affects the trajectories, but as in case (a), the net effect is that the height of trajectories at the exit is smaller than at the entrance $y_{f}<y_{i}$.

The case of high frequencies (case (c)) shows trapping, as for the longitudinal fields. The positive DEP on the particles make them move to the cylinder, and there are trajectories that end on the cylinder surface so that they can be trapped. The difference with the case of the longitudinal field is that the high-amplitude positions of the electric field are shifted $90^{o}$.

### 3.3. Particle Deviations

**Figure 5:**
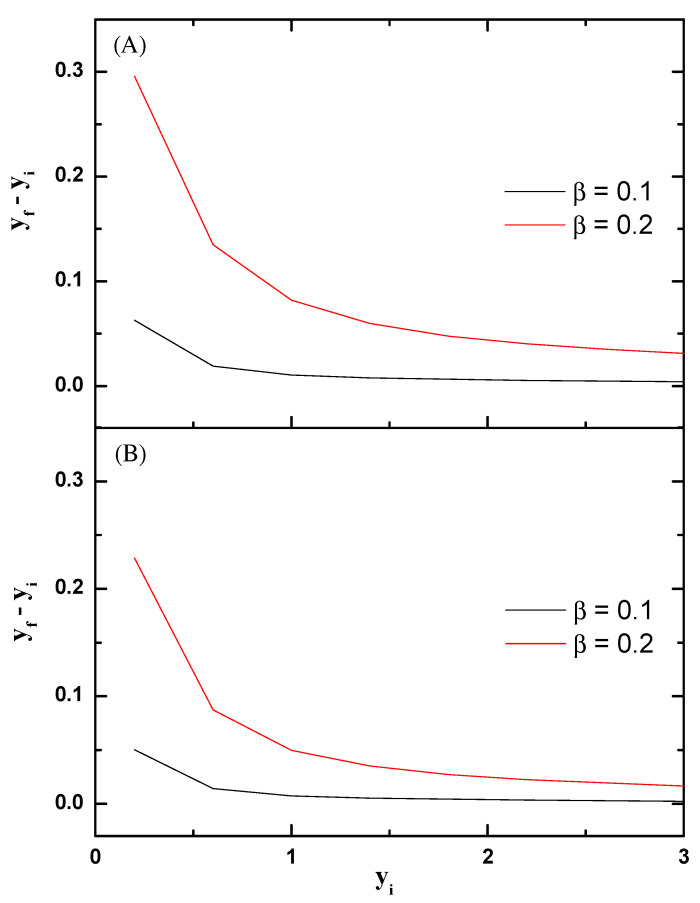
Deviation $y_{f}-y_{i}$ versus $y_{i}$ at N=50 for two cases: (**A**) low frequency and Λ=1; (**B**) low frequency and Λ=0.5.

From the perspective of employing the particle-post interactions for separation, the cases analyzed at low frequency with the longitudinal field seem more promising, since they lead to $y_{f}>y_{i}$, i.e., greater particle deviation. At the least, producing greater particle deviation is pursued in the case of DLD devices where tunable particle repulsion from the posts is the objective [3]. In Figure 5, we show $y_{f}-y_{i}$ versus $y_{i}$ for cases (a) and (b) of Figure 2, that is, particle trajectories when the electric field is longitudinal with N=50 in the cases (a) Λ=1 and (b) Λ=0.5. In order to check the accuracy of the numerical calculations, we have repeated the simulations with meshes of increasing quality and checked that the results for the deviation converge to the data shown in Figure 5.

## 4. Conclusions

In this paper, we have studied the scattering of a colloidal metal particle by an insulating circular post when subjected to an AC electric field. The particles are driven by fluid flow, and deviations from their original trajectory are studied as a function of the applied AC field. The forces that we have considered in this study are the dielectrophoresis and dipolophoresis forces, the wall repulsion by the image dipole, and the wall interaction by the ICEO flow reflected by the post. The latter two cases have been implemented using known expressions for plane walls. In the appendices, we have shown that these expressions are good approximations for small distances between the particle and the cylinder surface.

The relative influence of the forces on particle motion is discussed as a function of frequency of the AC field, particle size and distance to the post. We perform numerical simulations of the scattering of the metal colloid by the insulating circular post flowing in a microchannel. Our simulations show that the maximum particle deviation is found for an applied electric field parallel to the flow direction. Our theoretical predictions could be checked against experiments in microfluidic devices with suspensions of metal colloids or any other conducting particles such as colloidal conductive polymers. Additionally, greater deviations are found at low electric field frequencies, corresponding to the regime in which the ICEO interaction with the post is predominant over other mechanisms.

## Figures and Tables

**Figure A1:**
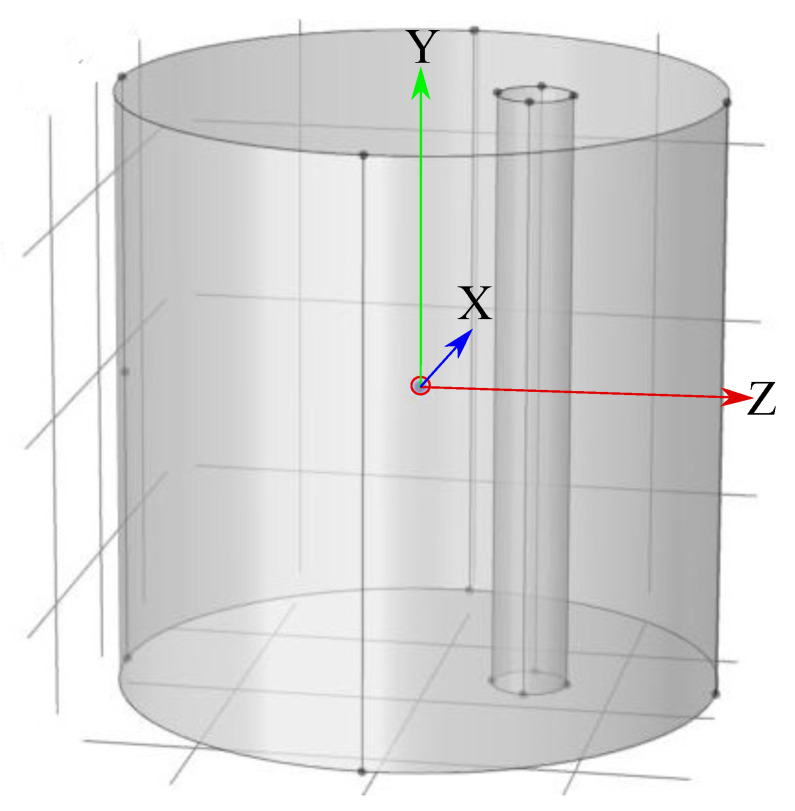
3D domain for computing the reflections by the cylinder of both the electric fields of the dipoles and the velocity field of the stresslets.

**Figure A2:**
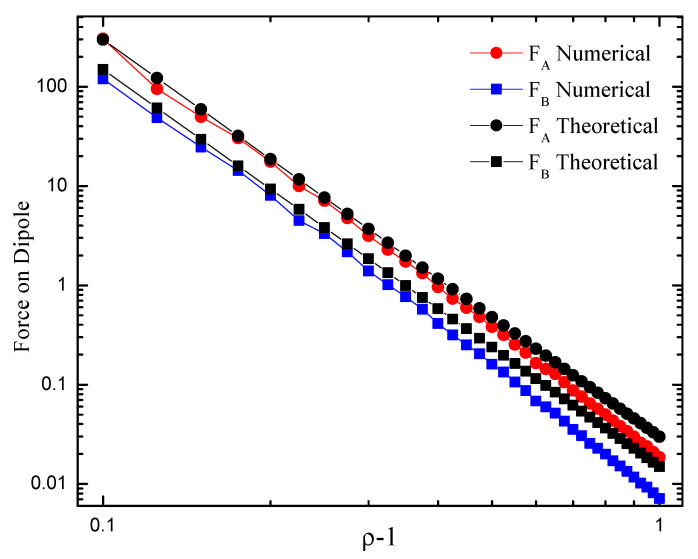
Nondimensional force on dipole versus distance to the cylinder surface. Comparison between numerical force and the theoretical approximation according to Equation (1).

**Figure A3:**
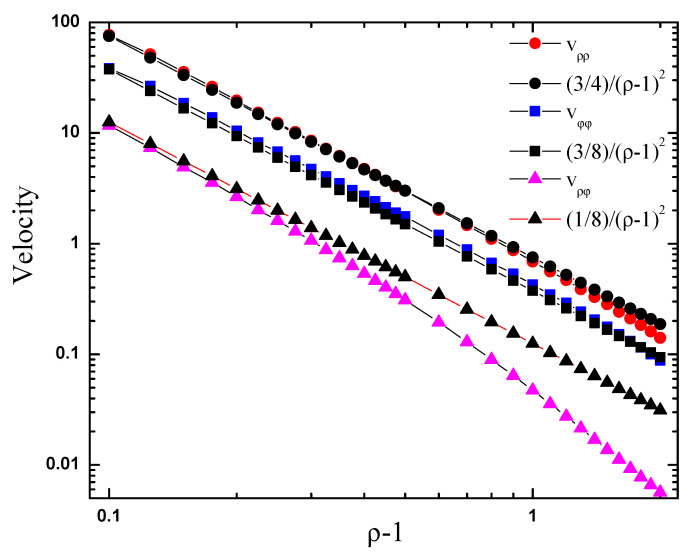
Particle drift velocity due to ICEO flow reflected on a cylinder versus the distance to the cylinder wall.

## Appendix A. Dipole-Dipole Repulsion from a Cylinder

In Section 2.1, the repulsion force on a dipole due to the presence of an insulating cylinder was approximated using the force due to an insulating plane wall. In this way, we obtained the drift velocity given by (4). Here, we check numerically the validity of this approximation. The potential due to a dipole in the presence of the insulating cylinder can be written as $\phi_{\mathrm{net}}=\phi_{\mathrm{dipole}}+\phi_{\mathrm{reflected}}$, where

(A1) $$\phi_{\mathrm{dipole}}=\frac{p\cdot r}{4\pi r^{3}}$$

and $\phi_{\mathrm{reflected}}$ is the potential reflected by the wall that results as a consequence of the boundary condition on the cylinder surface $\partial \phi_{\mathrm{net}}/\partial n=0$. Thus, $\phi_{\mathrm{reflected}}$ is a solution of Laplace’s equation with the boundary condition $\partial \phi_{\mathrm{reflected}}/\partial n=-\partial \phi_{\mathrm{dipole}}/\partial n$. We have solved this problem numerically with COMSOL Multiphysics in a 3D domain (see Figure A1). The computed force on the dipole $F=\left(p\cdot \nabla \right)E_{\mathrm{reflected}}$ can be compared to the approximation given by (4).

Figure A2 shows the numerical results for the force on a dipole as a function of the distance to the surface of the cylinder, ρ−1. The force is calculated for two different orientations of the dipole: (A) a dipole parallel to the radial direction $\hat{\rho }$ ($F_{A}$) and (B) a dipole perpendicular to the cylinder axis and to the radial direction $\hat{\rho }$ ($F_{B}$), i.e., when the dipole is oriented in the direction of $\hat{\varphi }$. For checking the flat-wall force approximation, Figure A2 also shows the forces calculated according to Equation (1), which results in the following expressions for the two orientations: $F_{A}=3/2\pi {\left(\rho -1\right)}^{4}$ and $F_{B}=3/4\pi {\left(\rho -1\right)}^{4}$.

## Appendix B. ICEO Interaction with a Cylinder

Because there is ICEO flow around the particle, we have assumed in Section 2.2 that the particle’s interaction with the post results in a drift velocity given by Equation (9), which corresponds to the particle drift velocity due to a flat wall. In this appendix, we check that this is a good approximation for small particles not far from the cylinder wall.

The ICEO flow given by Equation (5) coincides with the flow due to a stresslet for r≫a (Equation (7)). The flow field of the stresslet becomes affected by the presence of a post. The net flow field can be written as $v_{\mathrm{net}}=v_{\mathrm{stresslet}}+v_{\mathrm{reflected}}$, where $v_{\mathrm{reflected}}$ is the field generated by the wall of the post, which arises as a consequence of the no-slip boundary condition on the cylinder surface ($v_{\mathrm{net}}=0$). Thus, $v_{\mathrm{reflected}}$ is solution of the Stokes Equation (15) in a domain containing a cylinder of radius *R* with the following boundary condition on the cylinder wall: $v_{\mathrm{reflected}}=-v_{\mathrm{stresslet}}$. The velocity field $v_{\mathrm{reflected}}$ at the position of the stresslet (say $r_{0}$) is the drift velocity of the particle due to the presence of the nonslip cylinder wall ($u_{\mathrm{ICEO}}=v_{\mathrm{reflected}}\left(r_{0}\right)$). We are going to check that $u_{\mathrm{ICEO}}$ as given by Equation (9) is a good approximation by comparing with the numerical results.

For convenience, we write Equation (7) as:

(A2) $$\frac{v_{\mathrm{stresslet}}}{Ua^{2}}=p_{x}^{2}v_{xx}+p_{z}^{2}v_{zz}-6p_{x}p_{z}v_{xz}$$

where $p_{x},p_{y},p_{z}$ are the Cartesian coordinates of a unit vector in the direction of the electric field, and we have defined the following components of the stresslet field:

(A3) $$\begin{array}{ccc} v_{xx} & = & \left(\frac{1}{r^{3}}-\frac{3x^{2}}{r^{5}}\right)\hat{r} \end{array}$$

(A4) $$\begin{array}{ccc} v_{zz} & = & \left(\frac{1}{r^{3}}-\frac{3z^{2}}{r^{5}}\right)\hat{r} \end{array}$$

(A5) $$\begin{array}{ccc} v_{xz} & = & \left(\frac{xz}{r^{5}}\right)\hat{r} \end{array}$$

Placed at the stresslet position, we take $-\hat{z}$ as $\hat{\rho }$, the direction from the center of the cylinder to the stresslet, and $\hat{x}$ as $\hat{\varphi }$, the direction perpendicular to the cylinder axis and to $\hat{\rho }$. We have used COMSOL Multiphysics in the 3D domain previously shown to find $v_{\mathrm{reflected}}$ for each of the three stresslet components (A3) as a function of the distance to the cylinder axis. Figure A3 shows the comparison between the reflected velocity due to a cylindrical wall calculated numerically with the reflection calculated using Equation (9).

## References

[1] Morgan H., Green N.G. (2003). AC Electrokinetics: Colloids and Nanoparticles.

[2] Beech J.P., Jönsson P., Tegenfeldt J.O. (2009). Tipping the balance of deterministic lateral displacement devices using dielectrophoresis. Lab Chip.

[3] Calero V., Garcia-Sanchez P., Honrado C., Ramos A., Morgan H. (2019). AC electrokinetic biased deterministic lateral displacement for tunable particle separation. Lab Chip.

[4] Ho B.D., Beech J.P., Tegenfeldt J.O. (2020). Charge-Based Separation of Micro-and Nanoparticles. Micromachines.

[5] Calero V., Garcia-Sanchez P., Ramos A., Morgan H. (2019). Combining DC and AC electric fields with deterministic lateral displacement for micro-and nano-particle separation. Biomicrofluidics.

[6] Frechette J., Drazer G. (2009). Directional locking and deterministic separation in periodic arrays. J. Fluid Mech..

[7] van de Ven T.G., Warszynski P., Wu X., Dabros T. (1994). Colloidal particle scattering: A new method to measure surface forces. Langmuir.

[8] Wu X., Van de Ven T. (1996). Characterization of hairy latex particles with colloidal particle scattering. Langmuir.

[9] Whittle M., Murray B.S., Dickinson E. (2000). Simulation of colloidal particle scattering: Sensitivity to attractive forces. J. Colloid Interface Sci..

[10] Lapizco-Encinas B.H., Simmons B.A., Cummings E.B., Fintschenko Y. (2004). Insulator-based dielectrophoresis for the selective concentration and separation of live bacteria in water. Electrophoresis.

[11] Lapizco-Encinas B.H., Ozuna-Chacón S., Rito-Palomares M. (2008). Protein manipulation with insulator-based dielectrophoresis and direct current electric fields. J. Chromatogr. A.

[12] Lapizco-Encinas B.H. (2019). On the recent developments of insulator-based dielectrophoresis: A review. Electrophoresis.

[13] Pesch G.R., Du F., Schwientek U., Gehrmeyer C., Maurer A., Thöming J., Baune M. (2014). Recovery of submicron particles using high-throughput dielectrophoretically switchable filtration. Sep. Purif. Technol..

[14] Lorenz M., Malangré D., Du F., Baune M., Thöming J., Pesch G.R. (2020). High-throughput dielectrophoretic filtration of sub-micron and micro particles in macroscopic porous materials. Anal. Bioanal. Chem..

[15] Beech J.P., Keim K., Ho B.D., Guiducci C., Tegenfeldt J.O. (2019). Active posts in deterministic lateral displacement devices. Adv. Mater. Technol..

[16] Fernández-Mateo R., Calero V., Morgan H., García-Sánchez P., Ramos A. (2022). Wall Repulsion of Charged Colloidal Particles during Electrophoresis in Microfluidic Channels. Phys. Rev. Lett..

[17] Rose K.A., Hoffman B., Saintillan D., Shaqfeh E.S., Santiago J.G. (2009). Hydrodynamic interactions in metal rodlike-particle suspensions due to induced charge electroosmosis. Phys. Rev. E.

[18] Katzmeier F., Altaner B., List J., Gerland U., Simmel F.C. (2022). Emergence of Colloidal Patterns in ac Electric Fields. Phys. Rev. Lett..

[19] Cummings E.B., Singh A.K. (2003). Dielectrophoresis in microchips containing arrays of insulating posts: Theoretical and experimental results. Anal. Chem..

[20] Jones T.B. (1995). Electromechanics of Particles.

[21] García-Sánchez P., Ren Y., Arcenegui J.J., Morgan H., Ramos A. (2012). Alternating Current Electrokinetic Properties of Gold-Coated Microspheres. Langmuir.

[22] Ramos A., García-Sánchez P., Morgan H. (2016). AC electrokinetics of conducting microparticles: A review. Curr. Opin. Colloid Interface Sci..

[23] Bazant M.Z., Squires T.M. (2004). Induced-charge electrokinetic phenomena: Theory and microfluidic applications. Phys. Rev. Lett..

[24] Gamayunov N.I., Murtsovkin V.A., Dukhin A.S. (1986). Pair interaction of particles in electric field. 1. Features of hydrodynamic interaction of polarized particles. Colloid J. USSR (Engl. Transl.).

[25] Oren S., Frankel I. (2020). Induced-charge electrophoresis of ideally polarizable particle pairs. Phys. Rev. Fluids.

[26] Saintillan D. (2008). Nonlinear interactions in electrophoresis of ideally polarizable particles. Phys. Fluids.

[27] Saintillan D., Darvel E., Shaqfeh E. (2006). Hydrodynamic interactions in the induced-charge electrophoresis of colloidal rod dispersionss. J. Fluid Mech..

[28] García-Sánchez P., Arcenegui J.J., Morgan H., Ramos A. (2015). Self-assembly of metal nanowires induced by alternating current electric fields. Appl. Phys. Lett..

[29] Yariv E. (2009). Boundary-induced electrophoresis of uncharged conducting particles: Remote wall approximations. Proc. R. Soc. Math. Phys. Eng. Sci..

[30] Smart J.R., Leighton D.T. (1991). Measurement of the drift of a droplet due to the presence of a plane. Phys. Fluids Fluid Dyn..

[31] Blake J. (1971). A note on the image system for a stokeslet in a no-slip boundary. Mathematical Proceedings of the Cambridge Philosophical Society.

[32] Shilov V., Simonova T. (1981). Polarization of electric double-layer of disperse particles and dipolophoresis in a steady (DC) field. Colloid J. USSR.

[33] Miloh T. (2008). A unified theory of dipolophoresis for nanoparticles. Phys. Fluids.

[34] Miloh T. (2008). Dipolophoresis of nanoparticles. Phys. Fluids.

[35] Flores-Mena J.E., García-Sánchez P., Ramos A. (2020). Dipolophoresis and Travelling-Wave Dipolophoresis of Metal Microparticles. Micromachines.

[36] Huang L.R., Cox E.C., Austin R.H., Sturm J.C. (2004). Continuous particle separation through deterministic lateral displacement. Science.

[37] Kim S.C., Wunsch B.H., Hu H., Smith J.T., Austin R.H., Stolovitzky G. (2017). Broken flow symmetry explains the dynamics of small particles in deterministic lateral displacement arrays. Proc. Natl. Acad. Sci. USA.

[38] Lyklema J. (1995). Fundamentals of Interface and Colloid Science.

[39] Calero V., Fernández-Mateo R., Morgan H., García-Sánchez P., Ramos A. (2021). Stationary Electro-osmotic Flow Driven by ac Fields around Insulators. Phys. Rev. Appl..

[40] Calero V., Garcia-Sanchez P., Ramos A., Morgan H. (2020). Electrokinetic biased Deterministic Lateral Displacement: Scaling Analysis and Simulations. J. Chromatogr. A.

